# A millimeter-scale insight into formation mechanism of lacustrine black shale in tephra deposition background

**DOI:** 10.1038/s41598-022-15715-4

**Published:** 2022-07-07

**Authors:** Senhu Lin, Lianhua Hou, Xia Luo, Yiwen Wu

**Affiliations:** 1 Department of Oil and Gas Geology, Research Institute of Petroleum Exploration and Development, PetroChina, Xueyuan Road 20, Beijing, 100083 China; 2 Central Laboratory of Geological Science, Research Institute of Petroleum Exploration and Development, PetroChina, Xueyuan Road 20, Beijing, 100083 China; 3 School of Energy Resource, China University of Geosciences, Xueyuan Road 29, Beijing, 100191 China

**Keywords:** Palaeoclimate, Geochemistry, Petrology

## Abstract

To reveal the role of tephra in the deposition of black shale during periods of volcanic activity, we performed lithostratigraphic and geochemical analyses on 14 horizontally sliced samples drilled from a 2-cm-thick black shale interval in the lower Ch7 Member of the Upper Triassic Yanchang Formation, southern Ordos Basin. Results indicate that fewer plankton is preserved during tephra deposition than during periods of volcanic quiescence. With the decrease of volcanic activities and tephra deposition, the abundance of redox-sensitive trace elements (RSTEs) and biolimiting elements increases, while terrigenous elements gradually decrease, resulting in the improvement of organic matter (OM) preservation. Paleoenvironmental proxies suggest that the climate during the Late Triassic was generally warm and humid. However, subsequent intense volcanic eruptions may have caused climatic cooling that affected the water column, resulting in enhanced salinity, primary production, water stratification, and bottom water anoxia, leading to enhanced organic carbon production and preservation. Primary productivity and redox conditions controlled the accumulation of organic carbon. Although physical and chemical reactions relating to the deposition of tephra into water are short-lived, climate change induced by volcanic eruptions is the critical cause of black shale formation.

## Introduction

The "shale gas revolution" has driven the world's unconventional oil and gas exploration^1^. Shale oil resources have also become the focus of the petroleum industry. According to the U.S. Energy Information Administration^2^, the global recoverable shale oil resources are 469 × 10^8^ t. In 2019, the U.S. shale oil production was 3.74 × 10^8^ t, accounting for 66% of the U.S. crude oil production^3^. China is rich in shale oil resources, and it is estimated that the technically recoverable shale oil resources will reach 145 × 10^8^ t^4^. As the mining technology becomes more mature and perfect, shale oil is expected to become an essential strategic replacement resource for China's petroleum supply.

The Ch7 Member of the Upper Triassic Yanchang Formation in the Ordos Basin, China is a set of shale oil resources with ultra-high organic matter (OM) abundance (average TOC is 11%, the highest TOC is 35%)^5,6^. It is of great significance in investigating the formation process and genetic mechanism of such organic-rich shales. Additionally, the product of the intense volcanic activity, tephra, is commonly associated with the black shales in the Ordos Basin, which suggests the tuff interlayers might impact the organic carbon abundance in such shales^7^. Some significant biotic and environmental changes occurred during the Triassic–Jurassic transition, including the extinction of 76% of species^8–12^, long periods of rainfall^13^, and severe hypoxia^14,15^. The causal relationships and the mutual response degree of these events have been global research foci in recent years. Evidence for volcanic eruptions (namely tephra layers) during the Triassic–Jurassic transition is widespread in Europe and northern China^7,16^. Shale deposits in the Upper Triassic Yanchang Formation (Ordos Basin, northern China) preserve a continuous record of changes in OM enrichment during this period, and the tephra layers are well-preserved^17,18^. Thus, this region is ideal for investigating the relationship between tephra deposition and OM accumulation.

Previous studies have suggested that volcanic eruptions and tephra would enhance the preservation of organic matter in ancient black shales^7^. However, these studies did not decipher the role of tephra deposition in forming black shales. Since the shale change frequently within a small thickness range and exhibit strong heterogeneity^19,20^, in this study, the processes of OM formation and preservation within a short period (ca. 1000 years) before and after tephra deposition with the aid of millimeter-scale core sampling ware reconstructed. A longer-term analysis is not conducted because previous studies have confirmed that the effects of tephra emplacement on shale formation are short-lived^21,22^. We attempt to investigate the influence of tephra deposition on the organic matter enrichment with an example of the Yanchang Formation shales and reveal the formation mechanism of ancient black shales by analyzing the OM characteristics as well as the productivity, biological activity, redox conditions, and paleoenvironments in response to tephra deposition.

**Figure 1:**
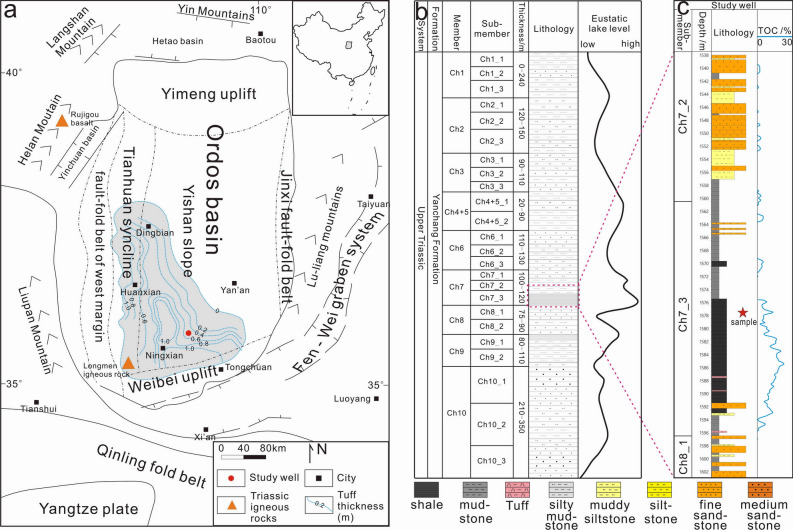
(**a**) The Upper Triassic Ordos Basin showing the distribution of tuff at the bottom of the Ch7 Member (data from Ref.^23^. Software: CorelDRAW Graphics Suite 2021, full 15-Day free trial. URL link: https://www.coreldraw.com/en/product/coreldraw/); (**b**) stratigraphy of the Upper Triassic Yanchang Formation (data from Refs.^25,26^). The lower Ch7 Member corresponds to the maximum flooding surface; (**c**) lithological profile of the studied well and sample location.

The Ordos Basin, located on the west of the North China Plate, is a large Mesozoic intracratonic sedimentary basin. The Cenozoic grabens near the basin are surrounded by mountains (Fig. 1a). The basin was opened from the Middle Triassic to the Early Cretaceous and has developed since the Late Cretaceous to its current size of ~ 25 × 10^4^ km^2^^23^. During the Middle-Late Triassic, the Ordos Basin was dominated by a fluvial and lacustrine environment, resulting in the deposition of the Yanchang Formation with a thickness of 800–1200 m. The Yanchang formation comprises a set of cyclical deposits of interbedded black shale, dark-gray mudstone, and gray siltstone that record the entire process of lake formation, expansion, shrinkage, and extinction in the basin^24^. The formation is divided into ten members (Ch1-Ch10) from top to bottom (Fig. 1b). The Ch7 Member is generally 95–140 m thick, which represents the greatest extent of the Triassic lake in the basin. In the sedimentary period of Ch7 Member, the lake area was larger than 5 × 10^4^ km^2^, and the water depth was up to 150 m^27^. The basin center is dominated by fine-grained deposits^28^, accompanied by numerous tuff layers. More than 180 tuff layers with thicknesses of 1 mm to a few centimeters have been found in the Ch7 Member^7^. As a volcanic material, tuff is intermediate acidic and is transported by air or water^29^. Tuff interbeds in the Ch7 Member are several millimeters to tens of centimeters thick, with a total thickness of up to 3 m^30^. The interbed gradually thins from the southwest to the northeast (Fig. 1a). Due to a lack of isotopic geochronology, the sedimentary age of the Ch7 Member remains controversial^31,32^. Tuffs, located in the middle and lower parts of the Ch7 Member, yield zircon U–Pb ages of 234 and 239.5 Ma, respectively^33^, indicating that deposition of the Ch7 Member began at 239.5 Ma and ended after 234 Ma, with a duration of at least 5.5 Ma. We selected a 2-cm core sample to reconstruct the petrological and geochemical evolution on a millimeter scale. The sample records a single deposition cycle (approximately 1000 years of duration) from lacustrine to tuff to lacustrine phases. The response characteristics of the microenvironment provide us with new insights into the formation mechanism of black shale in the tephra deposition background.

## Results

### Millimeter-scale mineral composition and lithofacies

**Figure 2:**
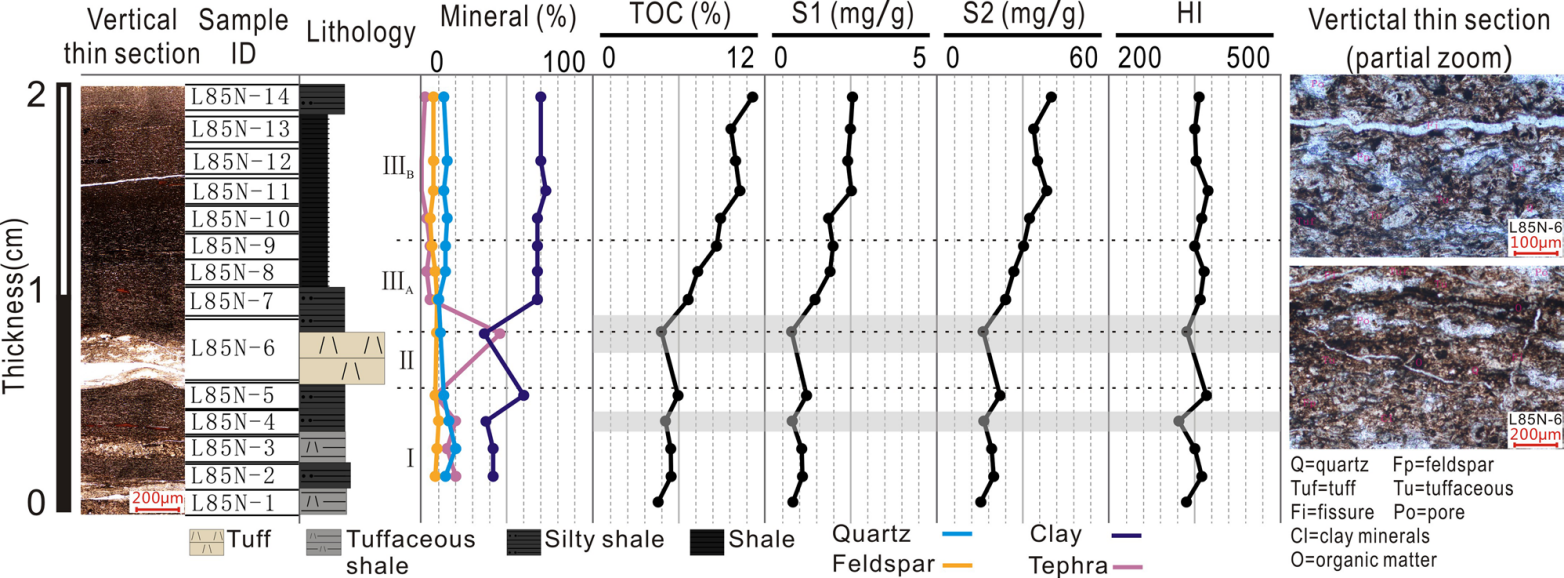
Lithology, mineral compositions, and geochemical indices of the studied samples. The vertical thin sections show the location of the tuff layer and 14 slices. The magnification of slice 6 confirms the structure and composition of tuff. S_1_ is the free hydrocarbon. S_2_ is the pyrolyzed hydrocarbon. HI is the hydrogen index.

Due to the organic matter enrichment, the vertical thin section in Fig. 2 is brown-yellow under the plane-polarized light. No visible fossils or sedimentary structures can be observed. Slice 6 is a set of relatively thick (~ 3 mm) tuff (Fig. 2), mainly composed of tuffaceous and clay minerals. A small amount of feldspar, quartz, and tuff debris are scattered in Slice 6. Locally, the tuff is metasomatized by dolomite, while the intergranular pores are filled with mineral asphalt. Except for slice 6, other slices characterize black shales, although they differ slightly in mineral compositions. Slices 2–5 contain relatively more tuffaceous debris and interstitial filling than slices 7–14. Slices 7–14 contain larger organic debris, more mineral asphalt, and less tuffaceous materials than slices 1–6 (Fig. 2).

As shown in Fig. 2, the TOC content for 14 slices ranges from 4.54 to 11.2%. The TOC content increases with the decreasing burial depth. By comparing, in slices 1–4, TOC is stable at ~ 5%. TOC increases to ~ 6% in slice 5 before it drops back to 4.8% in slice 6 when the tuff band occurs. Subsequently, in slice 7, TOC rapidly increases up to 6.6%. TOC increases steadily in slices 7–9 and stabilizes at ~ 10% in slices 10–14. Overall, TOC in slices 1–5 is approximately half of that in slices 10–14 (Fig. 2). According to the tuff occurrence and TOC content, 14 slices can be divided into three categories: slices 1–5 record the initiation of tephra deposition (phase I: active stage), with a small amount of tephra preserved due to locally weak volcanic activity; slice 6 denotes the deposition during an active regional volcanic eruption (phase II: eruptive stage); and slices 7–14 indicate depositions after the cessation of volcanic activities (phase III: intermittent stage). Slices 7–9 are further classified as phase III_A_, as they exhibit gradually increasing TOC, whereas slices 10–14 are classified as phase III_B_ because their TOC contents are much higher (~ 10%). Additionally, according to the mineralogical analysis in Fig. 2, the average content of tuffaceous debris is 23% during phase I, and the tuffaceous material is dispersed amongst lacustrine shale. During phase II, the average content of tuffaceous debris is 47%, indicating strong volcanic activity. Entering phase III, the tuffaceous debris content shows a marked decrease (average 4%). The quartz content is relatively stable (~ 12%) throughout 14 samples, exhibiting little change before and after the volcanic activity. The clay mineral content increases significantly after tephra deposition (averaging 43% and 65% in slices 2–5 and 7–14, respectively).

### Geochemical characterizations

Figure 2 shows the free hydrocarbon (S_1_) and pyrolyzed hydrocarbon (S_2_) for 14 samples. In general, evolutions of S_1_ and S_2_ are similar to that of TOC. The average S_1_ values in phases I, II, III_A_, and III_B_ are 0.98, 0.76, 1.79, and 2.49 mg/g, respectively. The average S_2_ values in phases I, II, III_A_, and III_B_ are 18.47, 16.04, 28.39, and 36.92 mg/g, respectively. The TOC, S_1_, S_2_, and HI (hydrogen index) values are the smallest in slice 6, indicating the relatively low OM abundance and hydrocarbon generation potential within the tephra deposit.

### Organic petrology

**Figure 3:**
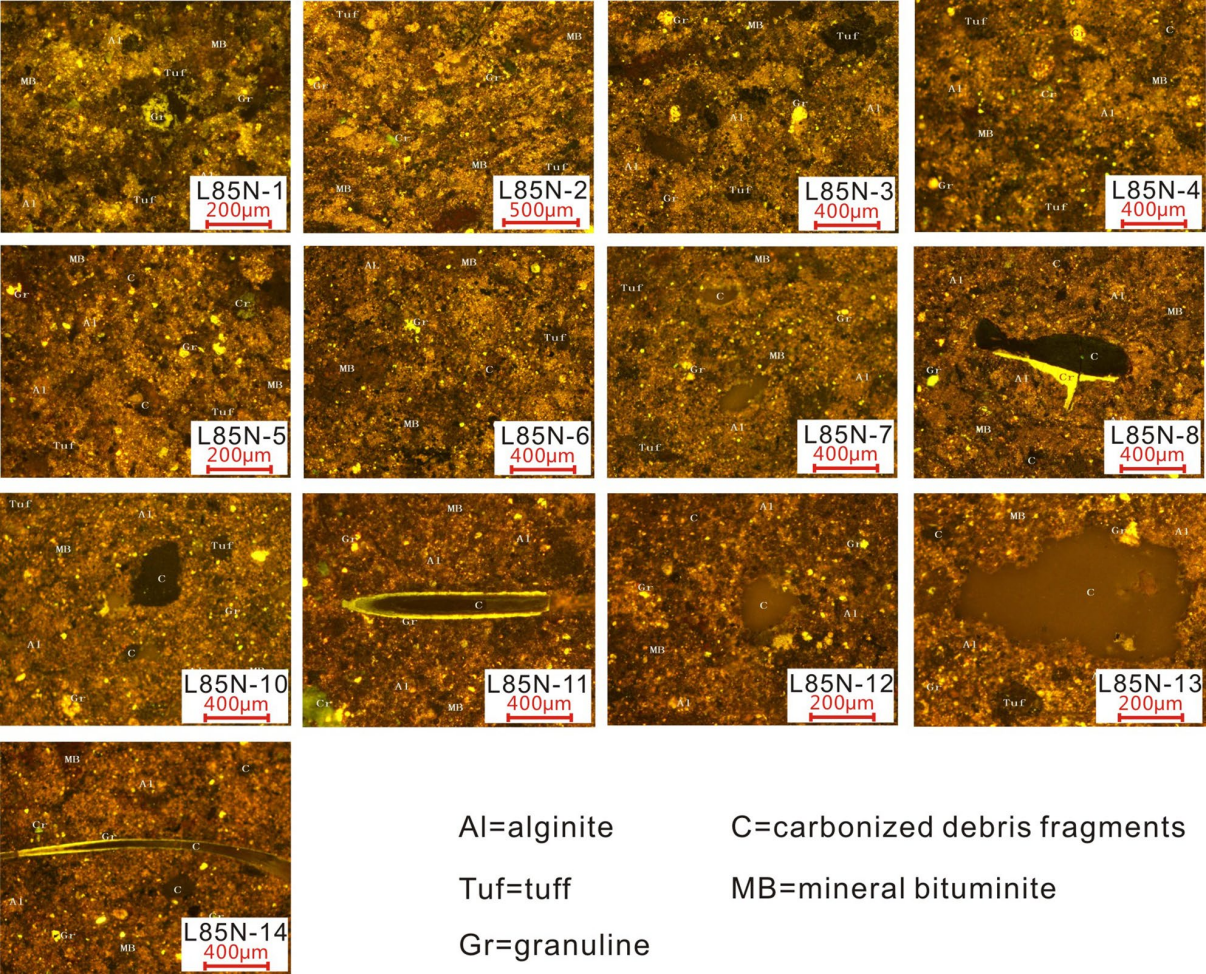
Kerogen macerals for 14 samples. Maceral photographs: L85N-1, alginite is acervate with brown-yellow fluorescence; the mineral bituminite has weak fluorescence as the base. L85N-2, alginite is densely distributed with scattered granuline and tuff debris. L85N-3, alginite is densely distributed. The particles are small, clastic, and enriched in liptinite that contains various sizes of uniformly distributed tuff debris. L85N-4, micro globular alginite nodules of the same shape and similar sizes are distributed, with more tuff debris. L85N-5, alginite is densely distributed in nodular form. L85N-6, the upper right of the image is part of a carbonized debris without fluorescence, and the lower right is alginite aggregates that are densely distributed, with a small amount of tuff debris. L85N-7, alginite is densely distributed and enriched in a nodular form. L85N-8 and alginite are distributed in a pelletoid form; single particles are fine with blurred shapes. L85N-10, alginite accounts for most of the OM and is densely distributed in groups. L85N-11, massive needle-columnar carbonized debris; alginite is widely distributed. L85N-12, alginite is the principal component of the OM. L85N-13, large and small carbonized debris fragments coexist; alginite is densely distributed. L85N-14, alginite is densely distributed and interbedded with scattered short bands of needle-columnar and granular carbonized debris fragments.

Moreover, kerogen macerals are shown in Fig. 3. Except for slice 6, maceral identification reveals that huminite and liptinite are significantly more abundant in phase III than in phase I, whereas vitrinite and inertinite are less abundant in phase III than in phase I. Therefore, the organic macerals can be classified into two categories. The first includes slices 1–5, which have similar characteristics. The tuff debris is coarse and widely distributed. The mineral bituminite matrix comprises a homogeneous mixture of tuff, clay minerals, and amorphous OM. Liptinite, especially microglobular alginites are relatively enriched in nodular forms. A large amount of inertinite, fusinite, and vitrinite occur (Table 1). The second category encompasses slices 7–14, generally characterized by the abundance of densely distributed alginite with blurry shapes. A small amount of fusinite and vitrinite occur (Table 1).

**Table 1:** Kerogen maceral data.

Sample no.	1	2	3	4	5	6	7	8	9	10	11	12	13	14
Huminite/%	7.7	6.4	8.9	6.7	13.6	4.3	11.7	10.2	11	10.7	8.6	10.7	9.2	14.1
Liptinite/%	53.4	55.7	57.2	55.3	53.2	45.8	62.5	64.6	63.1	71.7	70.3	66.7	58	66.1
Vitrinite/%	30.4	27.3	26.9	28.8	24.6	42.3	20.5	21.7	20.5	9.6	19	20.7	26.5	15.5
Inertinite/%	8.4	10.6	7	9.2	8.6	7.9	5.3	3.5	5.4	3	2.1	2.5	6.3	4.3

### Major terrigenous, biolimiting, and redox-sensitive trace elements

**Figure 4:**
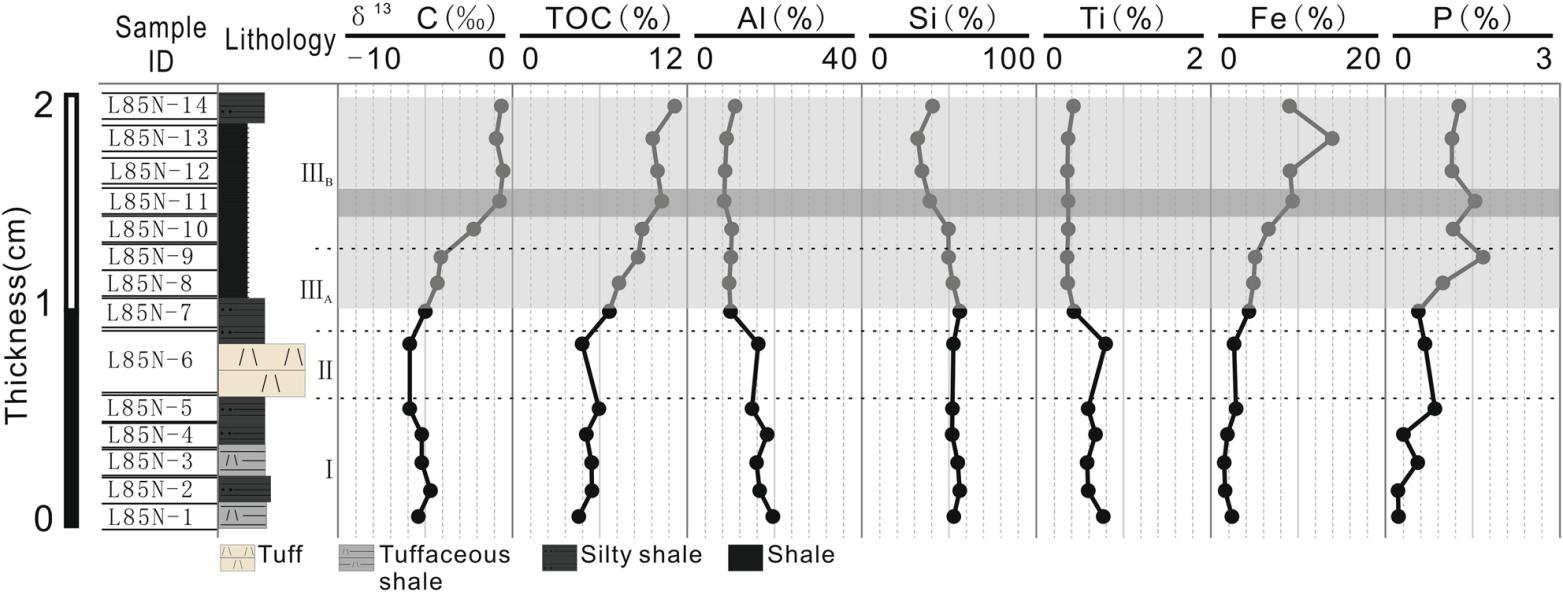
Carbon geochemical data (δ^13^C, total organic carbon (TOC)), major terrigenous elements (Si, Al, and Ti), biolimiting elements (Fe and P), and lithology for 14 samples. Enrichments in TOC are in gray; Si, Al, Ti, Fe, and P contents are in weight percentage.

**Figure 5:**
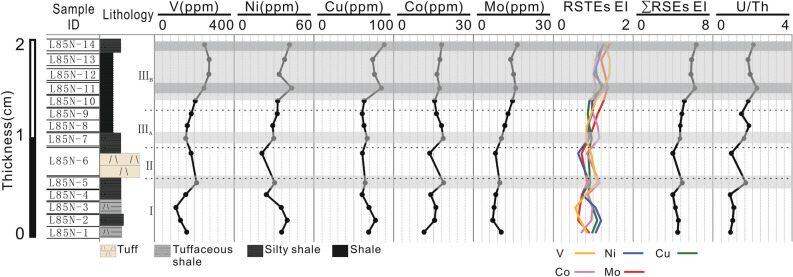
Lithology-related redox-sensitive trace elements (RSTEs), enrichment index values, ∑RSTEs enrichment index, and U/Th for 14 samples. RSTE enrichments are in grey bands; element abundances are in absolute ppm.

Figure 4 exhibits contents of the terrigenous elements (Al, Si, and Ti), carbon isotope (δ^13^C), and biolimiting elements Fe and P (i.e., elements present as components of DNA, RNA, polypeptides, ATP, phospholipids, and proteins that are involved in photosynthesis) for 14 samples. Overall, from bottom to top, the evolution of the terrigenous element is opposite to that of TOC and biolimiting elements. Aluminum (Al) content ranges from 8.36% (slice 11) to 19.64% (slice 1), with an average of 12.69% (Fig. 4). Silicon (Si) content ranges from 31.91% (slice 13) to 56.24% (slice 2), with an average of 48.14% (Fig. 4). Titanium (Ti) value ranges from 0.347% and 0.792% (average of 0.497%) (Fig. 4). Iron (Fe) values (average of 5.33%) are negatively correlated with Al (r = –0.78), Si (r =  − 0.93), and Ti (r =  − 0.71) values, but positively correlated with TOC (r = 0.88). Variations in P contents are similar to those in Fe, but P is enriched in slices 9 and 11. Carbon isotope values (δ^13^C) are strongly correlated with TOC contents (r = 0.94). Figure 5 shows the redox-sensitive trace elements (RSTEs) for 14 samples. All RSTEs exhibit four enrichment peaks, which are positively correlated with TOC (V: r = 0.81; Ni: r = 0.57; Co: r = 0.53; Cu: r = 0.60; and Mo: r = 0.95), and negatively correlated with terrigenous elements (e.g., Al, Ti). Additionally, each slice's relative elemental abundances are calculated to quantify their enrichments relative to the background level^34^. Compared with the background value, slices 5, 7, 11, and 14 are enriched in Co, for example, with *EIs* of 1.163, 1.157, 1.071, and 1.120, respectively. The ∑RSTEs *EI* (Fig. 5) ranges from 3.978 to 6.356, which is positively correlated with TOC (r = 0.94) and P (r = 0.72), and negatively correlated with the terrigenous elements (Al: r =  − 0.74; Si: r =  − 0.84; Ti: r =  − 0.71). The U/Th ratio ranges from 0.855 to 2.212, with an average of 1.483 (Fig. 5). The highest U/Th value occurs in slice 11 and positively correlates with TOC content (r = 0.89), P (r = 0.79), and Co (r = 0.74). However, U/Th is negatively correlated with Al (r =  − 0.88).

## Discussion

### Regional correlation between OM accumulation and tephra deposition

**Figure 6:**
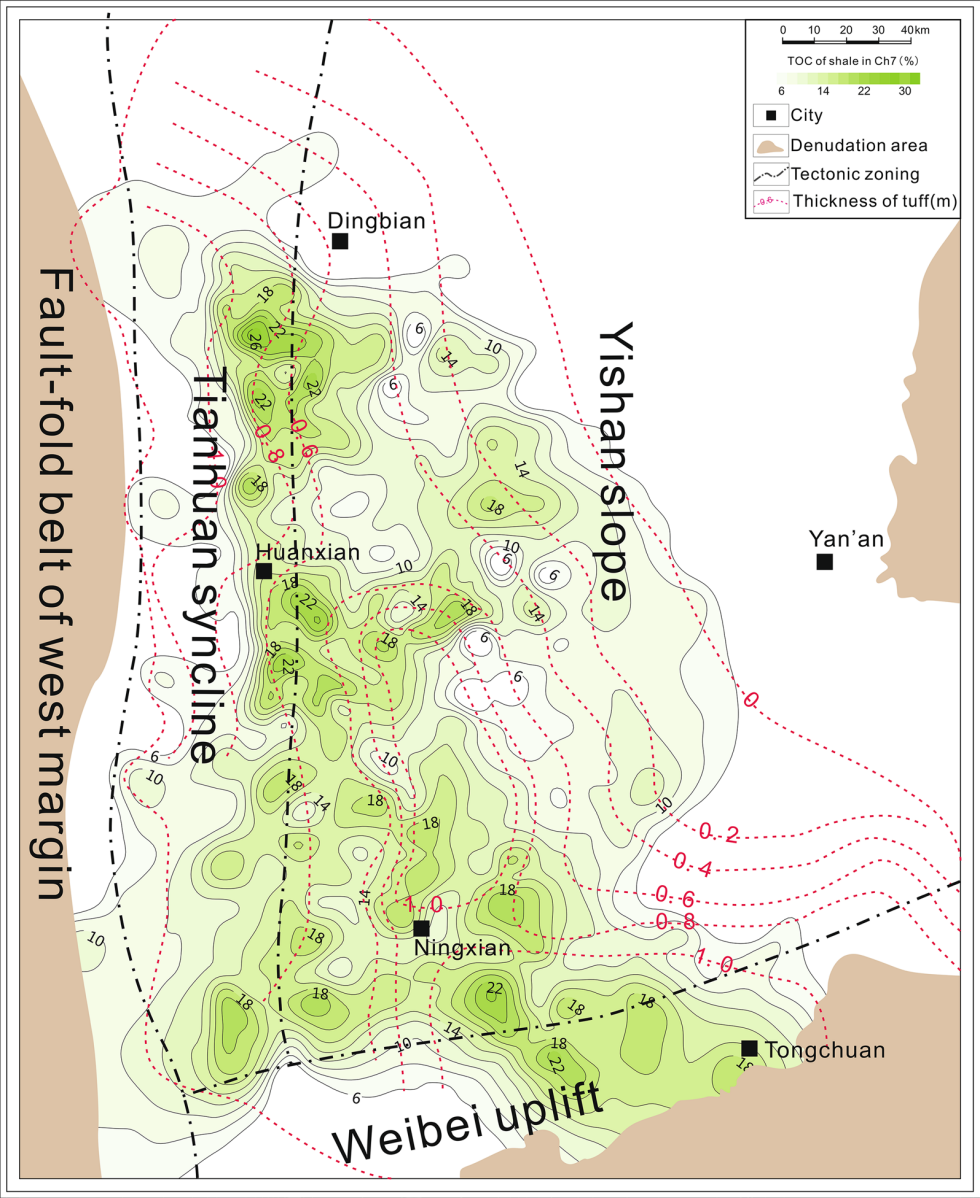
Distributions of accumulated tephra thicknesses and total organic carbon (TOC) in the Ch7 Member, Ordos Basin (Software: CorelDRAW Graphics Suite 2021, full 15-Day free trial. URL link: https://www.coreldraw.com/en/product/coreldraw/).

Previous studies concerning whether volcanic ash can promote the formation and preservation of organic carbon or not are controversial. Some studies recorded that tephra deposits promote the formation of OM by Fe fertilization^21,35^, oxidant exposure reduction^36^, and reactive oxide complexation^37,38^. However, some other studies suggested that tephra deposition and OM content are not closely related^39^. For example, regarding Fe fertilization in the open ocean from tephra deposition, the average chlorophyll levels return to pre-bloom values within three months of a volcanic event^21,22^. Furthermore, reactive oxide complexation induced by tephra deposition only produces an increase (~ 5%) in the total OM burial rate^40,41^. In the Ordos Basin, tuff layers with thicknesses ranging from a few millimeters to a few centimeters coexist with black shales in the Ch7 Member (Fig. 6)^30^. The region with high TOC does not entirely overlap with the areas of the thickest tuff (Fig. 6). Moreover, black shales with TOC greater than 15% (extremely organic-rich) are distributed throughout the tuff region, regardless of their thickness (Fig. 6). Studies on the relationship between OM enrichment and tephra occurrence have mostly been qualitative^18,42^. A preliminary conclusion is that tephra is conducive to forming black shales^7^. However, this conclusion is not supported by regional evidence because there is no positive correlation between the cumulative tuff thickness and TOC of shale at the basin scale (Fig. 6). Therefore, the deposition flux of tephra could not be the critical factor in promoting the formation and preservation of organic carbon. In addition, the strong heterogeneity of lacustrine deposits may weaken the genetic relationship between them. Our high-resolution results from a small-scale sample, extrapolated to a regional scale, are probably the best solution to indicate the relationship between the OM accumulation and tephra deposits.

### Millimeter-scale evidence of hypoxia influencing OM preservation

The abundance and depletion of different RSTEs, such as V, Ni, Co, Cu, and Mo, are valuable indicators for evaluating redox conditions in modern and ancient sedimentary systems^43–45^. The abundance of RSTEs in sediments relates to different reaction pathways under reducing conditions, leading to insoluble sulfides or organic metal complexes. Subsequently, products from the redox reaction are precipitated and adsorbed as oxides and hydroxides on the OM surface^34^. These elements enter lakes in various ways (e.g., through continental runoff) and share the properties of adjacent source rocks. As a result, abundant RSTEs provide sufficient evidence of anoxic conditions. Given that the redox indices might be contradictorily interpreted^46,47^, complementary auxiliary factors were used, such as TOC and RSTE enrichment index, to further validate results suggesting anoxic conditions. The U/Th ratio is used to assess redox conditions further. The oxygen depletion in the process of tephra deposition would decrease the abundance of soluble U in the water, resulting in U depletion in the water column and U enrichment in sediments^43,48–51^. Therefore, higher U/Th values might result from anoxic conditions. Additionally, high U/Th values of the slices are associated with organic-rich layers characterized by abundant RSTEs and high values of enrichment index (Fig. 5). Therefore, anoxic conditions are enhanced after the volcanic eruptions, and organic carbon is more effectively preserved. We observe increasing TOC under anoxic conditions in the absence of apparent lithologic change. Temporal variations in the RSTEs indicate fluctuating bottom water conditions. Our data show that the proxies (e.g., RSTEs) for oxygenation of the bottom water fluctuate in phases I, II, and III. Thus, the four intervals (slices 5, 7, 11, and 14) characterized by increasing values of RSTE enrichment index (Fig. 5) and the simultaneous occurrence of thin organic-rich layers indicate recurring episodes of hypoxia during this period.

### Reconstructions of temperature, water depth, and salinity

**Figure 7:**
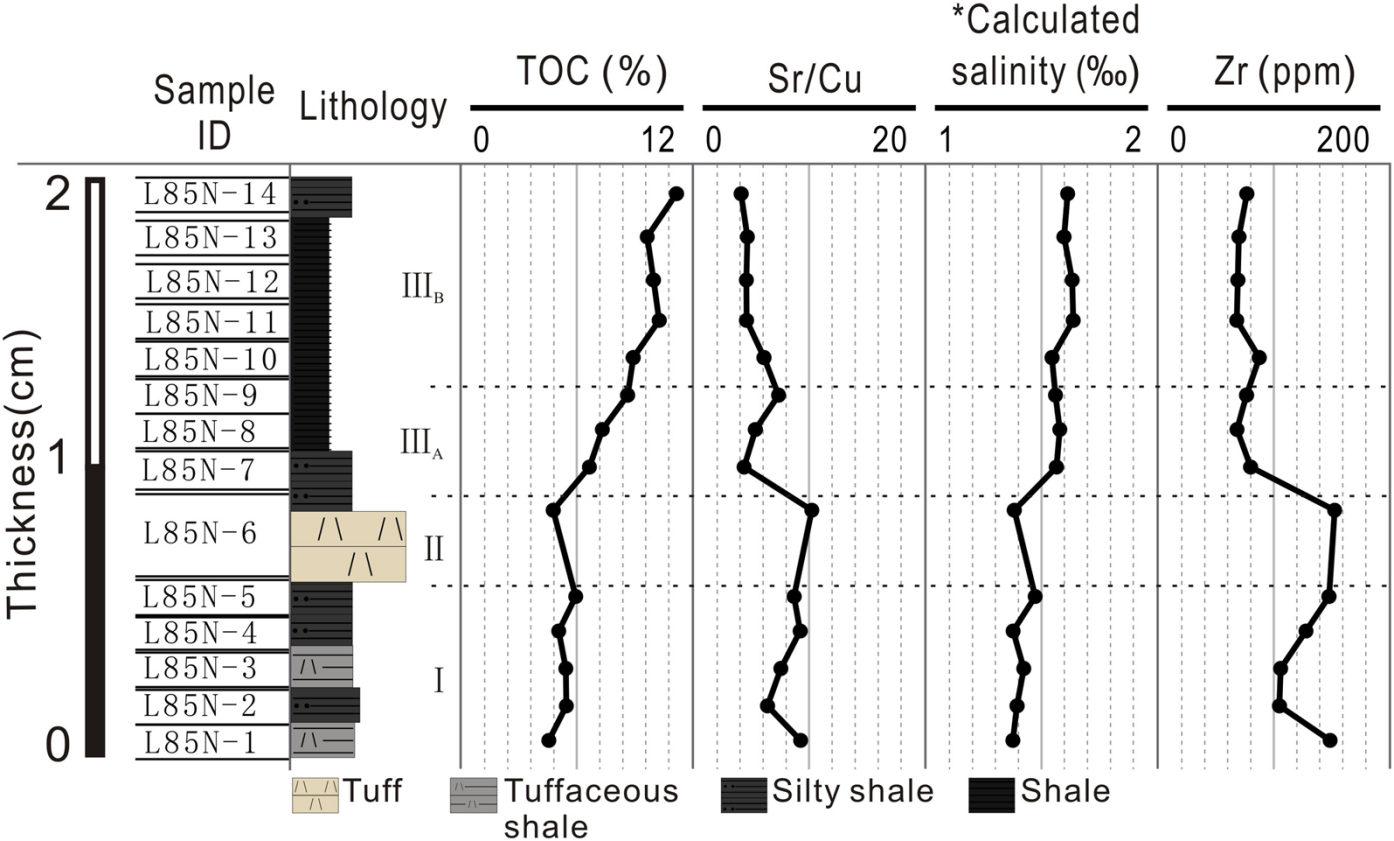
Zr element, calculated salinity, and Sr/Cu profiles for 14 samples, which could be used to indicate the palaeowater depth, paleosalinity, and paleotemperature, respectively. *The salinity was calculated based on equivalent boron content following the formula proposed by Couch^54^.

The contents and distributions of some elements are indicators of paleotemperature, paleowater depth, and paleosalinity. For example, Zr is a typical lithophile element (primarily mechanical migration) deposited near its source^52^. Thus, Zr is frequently used as an indicator of the transportation distance from a sediment source. Generally, Zr contents decrease with the increasing distance from the source. Boron can be used for quantitative reconstructions of salinity. Boron concentrations in marine and freshwater environments are generally 80–125 ppm and less than 60 ppm^53^, respectively. The formula proposed by Couch^54^ was used in this study, which has good applicability in continental strata, to calculate the paleosalinity of our samples. On average, in Fig. 7, the paleosalinity of the slices is ~ 1.5‰, indicating a freshwater environment. Consequently, as the climate during the studied interval becomes drier and colder, the paleowater depth decreases. The paleosalinity gradually increases with a small amount as the evaporation process exceeds the precipitation process. Sr/Cu values in lake sediments are sensitive to changes in climate. Values of 1–10 generally indicate warm and humid conditions, while those greater than 10 indicate hot and dry climates^55^. The Sr/Cu values of the samples (average of 6.59) correspond to a warm and humid environment. The values decrease from 9.26 in slice 1–4.09 in slice 14, indicating a decrease in paleotemperature. Except for an abnormally high value of 10.23 from slice 6, likely caused by elevated temperatures during volcanism, paleotemperatures gradually decrease with time in our samples (Fig. 7). In particular, Sr/Cu values decrease by ~ 50% after the cessation of volcanic activities because tephra can remain in the atmosphere to block the ultraviolet radiation and lower atmospheric temperatures^56^.

### Productivity and water stratification controlled OM preservation

The identification of organic macerals reveals the origin of OM. The OM in our slices is produced by a mixture of in situ and terrigenous photosynthates (Fig. 3). TOC fluctuations reflect environmental characteristics (Fig. 3) and a strong synchronization with peak P and Fe values (Fig. 4). P and Fe are key biolimiting elements for maintaining biomass productivity^57–61^. Our interpretation of the paleoenvironment depends on the known transport pathways of P and Fe to aquatic organisms, but their origins are unclear. During the sequestration of OM (Fig. 4), the decreasing relative abundance of terrigenous elements (Si, Al, and Ti) represents the decreasing river discharge and constrains factors controlling organic carbon production. Thus, rivers are not the primary contributor to the biolimiting elements (P and Fe); instead, they are supplied by other terrigenous sources containing heterogeneous OM (Fig. 4). A possible explanation is that the input of P and Fe into the lake water is relatively stable, but the concentrations of Si and Al are relatively high when the tephra is deposited, resulting in a dilution of P and Fe. Another possible explanation is that hydrothermal activity provides more P and Fe. However, there is insufficient evidence to confirm this scenario. During phase III, the primary productivity increases as the water becomes stratified and the biolimiting element contents (P and Fe) increase. Therefore, the continuous increase in biological oxygen demand in the water exacerbates the oxygen deficiency in the sediments, leading to enhanced OM preservation. Thus, the studied area reflects the influence of regional climate change. After the volcanic activity ceased, the water column is stratified due to the drier and cooler climatic conditions. Increasing paleosalinity in the organic-rich layers supports this inference (Fig. 7). The greater abundance of clay minerals in phase III (Fig. 2) may have provided favorable conditions for OM preservation by adsorption. Even when possible differences in the preservation conditions are considered, the boom of organisms after volcanic activities was significant, showing that nutrient levels were sufficient (Fig. 4). Previous studies have indicated a decrease in productivity with climates getting warmer. For example, the temperature increase affects the supply of nutrients to the euphotic layer, resulting in a decrease in phytoplankton productivity that cannot be offset by an increase in phytoplankton biochemical response rates^62^. For example, chlorophyll concentrations are low in the Pacific Ocean during warm periods^63^. Phytoplankton cell abundance in a cold-water area of the northeastern Atlantic Ocean increased, while that in a warm-water area decreased^64,65^ reported a negative correlation between temperature and the volume of phytoplankton cells: the volume decreased by 2.5%/°C, and the biomass of phytoplankton also decreased to some extent^66^. Consequently, the productivity increase shown by our data with decreasing temperature in the background of a warm, humid climate is reasonable.

### Black shale formation mechanism in tephra deposition background

Our results demonstrate that volcanic events would enhance the OM preservation and the formation of black shales^38^. Since biolimiting and redox-sensitive elements show strong correlations with TOC in the present study, the combined effects of productivity and hypoxia are the key factors controlling the formation and preservation of organic carbon. Increased RSTE contents, indicating redox conditions, primarily reflect enhanced water stratification. TOC enrichment is associated with relatively high values of paleosalinity (overall freshwater). Water with higher salinity would produce a stronger density gradient, causing more anoxic episodes. The direct impact of tephra deposition was transient and limited. The relatively long-term climate change caused by the volcanic eruptions might explain the enhanced OM accumulation in our samples. The largest tephra flux may have diluted the abundances of Fe and P, which were both sourced from terrigenous input. Intense volcanic eruptions resulted in a clear paleoclimatic cooling when the tephra entered the stratosphere. This would activate hypochlorous anhydride (ClO) through nitrate and sulfate, causing a sudden interdecadal decrease in total stratospheric ozone^56^. The reduction in absorbed heat would cause an interdecadal reduction in stratospheric temperature and geopotential height^56^. Under the warm and humid climate conditions in the Middle and Late Triassic, the Ordos Basin became colder due to tephra eruptions, further strengthening water-column stratification and enhancing TOC preservation. The cooler climate simultaneously enhanced the primary productivity of the phytoplankton in the water. Unlike modern oceans, tephra deposition did not directly promote the accumulation of organic carbon in the ancient lake. The fertilization of tephra has little contribution to the formation of the ancient thick organic-rich shale. Moreover, the extent of tephra deposition is influenced by airflow and topography. Its direct effect (e.g. reducing oxidant exposure) on the lacustrine sediments was probably local (Fig. 6). However, climatic, hydrologic, biological, and other environmental changes caused by volcanic activities were long-lasting and wide-ranging. They are the main contributing factors to organic carbon accumulation. This sedimentary model could provide a new understanding of the formation mechanisms of black shales.

## Conclusions

At a regional scale, the deposition flux of tephra does not influence the accumulation of organic carbon in the Ordos Basin during the Late Triassic. High-resolution stratigraphical and geochemical characteristics in a 2-cm shale from the lower Ch7 Member in the Ordos Basin were investigated at a millimeter scale. Depth profiles of the minerals, elements and organic components enable us to test possible deposition mechanisms for black shale in the tephra background. As suggested by biolimiting elements, high productivity would cause intense organic carbon deposition. During the dormant period, which is beneficial to OM deposition, the primary productivity is higher than that during the tephra deposition period. Geological factors controlling both booms and the extinctions of aquatic organisms in the lake basin influence OM abundance. Redox-sensitive elements indicate that oxygen levels persistently decreased in the lake bottom water along with the volcanic intermission, which would contribute to the high organic carbon content. In other words, a low oxygen content facilitates OM preservation. The physical and chemical reactions caused by tephra deposition are pretty short-lived. Therefore, the additional nutrients in surface water and anoxia in bottom water triggered by tephra deposition are difficult to record in the geological history. The variations of sedimentary environment recorded in shales resulted from climatic changes caused by tephra. A cooling climate for a considerable time after the eruption probably leads to increased salinity, primary productivity, water stratification, and bottom water anoxia, which promote the input and preservation of organic carbon on long timescales (ca. 1000 yrs). Conventional methods based on XRD analysis on pulverized samples provide average information of sedimentary beddings but may neglect distinct paleoenvironmental information. In this study, a system for lacustrine black shales to recover the information on individual bedding was established. To truly reflect the geological processes, integration of special geological events with the nutrient supply, the hydrologic state of lakes, and the flux of clastic sediments were carried out.

## Materials and methods

### Ch7 shale samples

The upper Triassic, Yanchang Formation, Ch7 Member Shale was deposited in the southern Ordos Basin, North China^5^. The thickness of the Ch7 Shale varies from 150 to ~ 10 m in Ordos Basin^27^, with ultra-high organic matter abundance^5,6^. The shale contains frequent volcanics thinning vertically from the bottom to top of the Ch7 Member^7^. Well L85 is located at the edge of the Yishan Slope (Fig. 1a). Samples collected from (Fig. 1c) sealed cores in Well L85 are mainly black shales with tuffaceous laminae. To determine the tephra effects on shale formation, it is necessary to investigate the sediments deposited before and after the tephra entered the paleolake water column. Given the deposition duration of the Ch7 Member was at least 5.5 Ma^31–33^, the sedimentation rate is less than 1.8–2.2 cm/ka. A 2-cm-thick shale core (101.6 mm diameter) sample containing a tuff layer (Fig. 2) was selected, which corresponds to an interval of ca. 1000 yrs. In addition, no tuffs exist at least 1 m above the selected sample, avoiding the influence of multiple tephra sets. A thousand years is much longer than the duration of a single volcanic eruption (several months)^21,22^.

### Sample processing

The 2-cm core sample was cut into two parts vertical to bedding (approximately 1:4 volume ratio). The small part was prepared as a large thin section vertical to bedding over the entire sampling range (leftmost image in Fig. 2). The larger part was slabbed and then sliced parallel to bedding at ~ 1 mm resolution, using a diamond wire saw. The 2-cm-thick sample was cut into 14 continuous slices (i.e., L85N-1 to L85N-14 from bottom to top, referred to as slices 1–14). The thickness of each slice ranges from 0.6 to 1.1 mm. Noticeably, slice 6 is a 3-mm-thick tuff. 4 cm^2^ segment of each slice was saved (in the same position) to prepare the transmission light and reflected light thin sections (horizontal thin section, slice 9 is absent due to damage). The rest part of each slice was respectively dried and powdered to 200 mesh, and subsamples were taken for individual analyses, including XRD, TOC, Rock–Eval, major and trace elements, and carbonate C-isotope.

### Facies identification via optical microscopy

Sample preparation followed ASTM D2797^67^ wherein the vertical slice was mechanically ground and polished by paper (120–1200 grit) followed by final polish on matte clothes with 1.0 μm alumina to achieve smooth surfaces for microscopic analysis. We took images using a transmitted light microscope (Olympus BX-53 manufactured by the Olympus Corporation, Tokyo, Japan) at the Central Laboratory of Geological Sciences (CLGS), PetroChina Research Institute of Petroleum Exploration and Development (Beijing, China). From the vertical thin section images, minerals and tuff layers are easy to be observed.

### Organic petrography analysis via optical microscopy

The 13 slices (except slice 9) were carried out organic petrography analysis by using maceral identification in epifluorescence followed ASTM D7708^68^. Maceral identification employed an Olympus BX53M microscope with LED illumination with the computer program PRECiV™ by the China University of Geoscience.

### Bulk characterization of shale

Total organic carbon (TOC) analysis and Rock–Eval were carried out at the CLGS following methods previously described in Barker^69^ and Peters et al.^70^ TOC were measured using a LECO CS-230 carbon–sulfur analyzer (LabX Corporation, Midland, Canada). Rock–Eval pyrolysis used a ROCK-EVAL6 pyrolyzer manufactured by Vinci Technologies, Nanterre, France, with about 60 mg of crushed powdered rock being heated using an initial oven program of 300 °C for 3 min and then from 300 to 650 °C at the rate of 25 °C min^−1^ in an N_2_ atmosphere. The oxidation stage was achieved by heating at 300 °C for 1 min and then from 300 to 850 °C at 20 °C min^−1^ and held at 850 °C for 5 min.

### Mineral composition determination

XRD measurements to determine mineralogy have been performed on Rigaku D/Max 2500 v/PC system using Cu Kα radiation (Rigaku Corporation, Tokyo, Japan) at the CLGS. Quantitative phase analysis is performed by using MDI Jade software. The precision of these measurements is better than 0.1wt% for phases in which the content is above 2%. Mineral compositions relate to the crystalline content of the analyzed samples.

### Major and trace element chemistry

Major and selected trace elements in samples rock powders were determined using an Axiom AX X-ray fluorescence spectrometer (Axiom Electronics LLC, Hillsboro, USA) and an ELEMENT XR inductively coupled plasma–mass spectrometer (Thermo Electron Corporation, Waltham, USA) at the Beijing Research Institute of Uranium Geology, China (BRUG). Internal precision and external reproducibility are typically better than 1% and 3%, respectively.

### Isotope ratio measurements

All samples were selected for isotope ratio measurements. The stable carbon isotope ratio of carbonate (δ^13^C_carb_) was measured on duplicate subsamples of 500 μg using an isotope ratio-mass spectrometer (Finnigan MAT-253, Thermo Electron Corporation, Waltham, USA) at the BRUG (China). The *δ*^13^C results are reported in delta notation concerning PeeDee Belemnite (PDB). The typical values were better than 0.06%.

### Statistical and multivariate analysis

The relative abundance for each element was estimated by the average value of the background level^34^ based on the following equation:

1 $${EI}_{A}=\frac{[{Element}_{A}]}{{[{Element}_{A}]}_{background}}$$

where $[{Element}_{A}]$ represents the concentration of each sample and ${[{Element}_{A}]}_{background}$ is the average value of the corresponding element for all the samples. EI means the enrichment index.

The correlation coefficient (r) of any two proxies is calculated by the Pearson's equation^71^:

2 $$\mathrm{r}(\mathrm{x},\mathrm{ y})=\frac{\sum (x-\overline{x})(y-\overline{y})}{\sqrt{\sum {(x-\overline{x})}^{2}\sum {(y-\overline{y})}^{2}}}$$

where x and y are the corresponding sample's average. The results of this equation go from a perfect negative correlation (− 1), no correlation (0) to a perfect positive correlation (1).

## Supplementary Information

Supplementary Tables.

## Data Availability

The raw datasets of this study are provided in the Supplementary Information, including five tables (supplementary Tables S1–S5).
