# Supplementary material for: A millimeter-scale insight into formation mechanism of lacustrine black shale in tephra deposition background
Source: Sci Rep. 2022 Jul 7;12:11511. doi: 10.1038/s41598-022-15715-4 (PMC9262940; doi:10.1038/s41598-022-15715-4)
Supplement: Supplementary file 1 — Supplementary Tables. [file 41598_2022_15715_MOESM1_ESM.pdf]

SUPPLEMENTARY INFORMATION

A millimeter-scale insight into formation mechanism of lacustrine black shale in tephra deposition background

Senhu Lin[1,*,+], Lianhua Hou[2,*,+], Xia Luo[1,*], Yiwen Wu[3]

[1]Department of Oil and Gas Geology, Research Institute of Petroleum Exploration & Development, PetroChina, Xueyuan Road 20, Beijing 100083, China

[2]Central Laboratory of Geological Science, Research Institute of Petroleum Exploration & Development, PetroChina, Xueyuan Road 20, Beijing 100083, China

[3]School of Ocean Science, China University of Geosciences, Xueyuan Road 29, Beijing 100191, China

*Corresponding authors: lsh2012@petrochina.com.cn; houlh@petrochina.com.cn; luoxia69@petrochina.com.cn.

[+]these authors contributed equally to this work.

Supplementary Table S1: Rock component content data.

Sample No.	*1*	*2*	*3*	*4*	*5*	*6*	*7*	*8*	*9*	*10*	*11*	*12*	*13*	*14*
Quartz (%)	/	11	16	12	8	10	7	9	11	10	9	10	/	9
Authigenic quartz (%)*	/	3	4	4	5	0	3	5	3	5	4	5	/	4
Feldspar (%)	/	8	9	10	8	8	9	8	6	5	7	7	/	7
Tephra (%)*	/	20	15	35	8	46	5	3	5	3	0	0	/	2
Clay mineral (%)	/	42	42	28	60	36	68	68	68	68	73	70	/	70
Pyrite (%)	/	5	5	6	8	0	7	6	6	7	6	7	/	7
Calcite (%)	/	11	9	5	3	0	1	1	1	2	1	1	/	1

* Authigenic quartz and tephra content was determined by mineral identification (thin section authentication).

Supplementary Table S2: TOC and Rock-Eval data.

Sample No.	*1*	*2*	*3*	*4*	*5*	*6*	*7*	*8*	*9*	*10*	*11*	*12*	*13*	*14*
TOC (wt. %)	4.54	5.45	5.43	5.06	5.94	4.77	6.66	7.33	8.65	8.94	10.30	10.00	9.67	11.20
Hydrogen index	335	363	350	322	371	336	360	367	350	363	373	353	350	358
S_1 (mg/g)	0.80	1.08	1.06	0.78	1.20	0.76	1.45	1.89	1.98	1.85	2.52	2.41	2.49	2.55
S_2 (mg/g)	15.23	19.76	19.03	16.31	22.03	16.04	23.96	26.88	30.29	32.41	38.47	35.26	33.87	40.08
S_3 (mg/g)	0.58	0.43	0.43	0.50	0.50	0.61	0.37	0.37	0.47	0.43	0.54	0.63	0.64	0.51

Supplementary Table S3: Major elements data.

Sample No.	*1*	*2*	*3*	*4*	*5*	*6*	*7*	*8*	*9*	*10*	*11*	*12*	*13*	*14*
Si (%)	52.77	56.24	55.05	51.83	52.01	52.55	56.24	52.27	49.73	49.65	38.87	34.36	31.91	40.47

Al (%)	19.64	16.57	15.87	18.33	14.82	16.26	9.86	9.52	9.94	10.12	8.36	8.6	8.93	10.88
Fe (%)	5.06	6.13	6.52	5.67	8.41	7.3	11.99	14.11	13.58	15.52	19.67	23.56	25.11	21.41
Mg (%)	1.53	1.22	1.24	1.35	1.54	1.63	1.53	1.72	1.77	2	2.14	2.76	2.96	2.39
Ca (%)	2.11	2.08	3.04	3.2	2.76	2.5	1.8	2.47	3.52	2.75	3.35	3.1	3.21	3.28
Na (%)	2.12	2.53	2.05	2.53	1.15	1.09	0.678	0.666	0.651	0.545	0.506	0.506	0.48	0.676
K (%)	3.28	2.42	2.47	2.77	2.64	2.98	1.81	1.74	1.84	1.92	1.62	1.65	1.7	2.02
Mn (%)	0.08	0.13	0.167	0.225	0.483	0.264	0.335	0.371	0.29	0.325	0.429	0.583	0.658	0.442
Ti (%)	0.765	0.591	0.578	0.676	0.59	0.792	0.426	0.353	0.347	0.36	0.358	0.35	0.358	0.42
P (%)	0.211	0.202	0.547	0.296	0.843	0.671	0.556	0.981	1.68	1.16	1.54	1.14	1.14	1.26
Lost (%)	12.43	11.7	12.29	12.97	14.73	13.84	14.54	15.6	16.29	15.07	23.13	23.37	23.37	16.16

Supplementary Table S4: Trace elements data.

Sample No.	***1***	***2***	***3***	***4***	***5***	***6***	***7***	***8***	***9***	***10***	***11***	***12***	***13***	***14***
Li (μg/g)	12.7	18.5	15	15.6	15.3	12.8	17.3	17.8	17.8	17.7	20	19.3	19.9	21.5
Be (μg/g)	2.44	1.85	1.9	2.1	2.48	2.53	1.76	1.88	1.95	2.31	2.53	2.48	2.72	2.83
Sc (μg/g)	21.5	15.7	17.5	17.9	24.3	19.3	12.9	14.8	17.1	14.5	13.4	11.4	11.6	11.6
V (μg/g)	162	130	107	157	212	184	157	165	185	205	249	274	276	253
Cr (μg/g)	124	133	134	137	146	93.2	98.1	87.9	81.1	70.1	70.9	57	54.7	56.4
Co (μg/g)	11.4	15.4	15.9	13.9	18.8	13.5	18.7	18	16.1	15.4	17.3	16.3	17.4	18.1
Ni (μg/g)	35.8	40	35.5	24.2	30.5	20.8	29.8	29	32.5	32.8	43.4	33.9	38	41.8
Cu (μg/g)	68.8	77.4	69.2	60.6	64.5	60.9	67	62.9	60.7	63.2	84.9	76.2	73.9	88.5
Zn (μg/g)	93.4	85.2	78.3	74.5	97.2	72.9	44.2	48.4	55.7	61.3	67.3	71.3	63.5	69.8
Ga (μg/g)	22.6	17.3	16.9	19	20.7	19.7	14	13.1	13.3	13.9	13.2	12.6	13.5	14.5
Rb (μg/g)	129	95.8	94.3	104	110	116	81.3	76.3	80.2	84.8	77.5	78.1	80.9	89
Sr (μg/g)	637	494	522	559	561	623	291	335	446	384	387	346	342	362
Y (μg/g)	20.5	16.2	32.9	22.6	49.3	41	23.8	30.6	34.2	25.2	20.6	19.4	20.9	23.8
Mo (μg/g)	10.4	7.19	7.7	8.37	9.84	8.21	10.5	11.8	13	14.7	15.9	15.1	14	16.5
Cd (μg/g)	0.375	0.329	0.299	0.313	0.403	0.32	0.265	0.273	0.359	0.358	0.411	0.4	0.373	0.484
In (μg/g)	0.089	0.074	0.069	0.071	0.081	0.072	0.048	0.048	0.055	0.058	0.05	0.053	0.05	0.061
Sb (μg/g)	1.89	2.4	2.51	2.08	2.93	2.21	3.49	3.56	3.25	3.27	4.83	3.41	3.34	3.56
Cs (μg/g)	14.5	10.2	9.71	11.1	11.7	12.9	7.82	7.04	7.33	8	7.11	7.33	7.37	8.23
Ba (μg/g)	782	628	613	663	661	681	405	412	474	447	420	410	428	460
La (μg/g)	16.1	17.1	32.1	21.5	50.6	41.8	33	38.6	35.6	27.1	24.2	23.5	25.4	29.3
Ce (μg/g)	34.2	38.1	59.9	43.6	94.8	78.4	60.6	66	59.5	48.1	43.7	43.6	45.4	52.2
Pr (μg/g)	4.14	4.68	7.26	5.3	11.5	9.33	7.3	7.86	7.09	5.82	5.2	5.09	5.47	6.31
Nd (μg/g)	16.5	18.9	28.5	21.5	45.9	36.4	28.1	30.9	27.6	22.6	20.4	19.6	21.5	24.4
Sm (μg/g)	3.35	3.84	5.77	4.22	9.09	6.95	5.31	5.82	5.3	4.34	3.76	3.72	4.01	4.66
Eu (μg/g)	0.67	0.673	1.27	0.887	2.09	1.44	1.02	1.2	1.03	0.771	0.715	0.692	0.769	0.908
Gd (μg/g)	2.84	3.02	5.08	3.49	7.87	6.21	4.66	5.32	4.97	3.85	3.44	3.34	3.66	4.2
Tb (μg/g)	0.537	0.542	0.917	0.667	1.43	1.13	0.78	0.894	0.843	0.689	0.584	0.561	0.603	0.718
Dy (μg/g)	3.32	3.05	5.11	3.82	7.93	6.36	4.09	4.74	4.81	3.81	3.16	3.02	3.28	3.82
Ho (μg/g)	0.781	0.636	1.12	0.846	1.67	1.38	0.829	1.03	1.05	0.843	0.679	0.639	0.709	0.801
Er (μg/g)	2.22	1.74	2.91	2.34	4.33	3.65	2.23	2.74	2.84	2.27	1.86	1.74	1.91	2.15

Tm (μg/g)	0.482	0.353	0.56	0.485	0.82	0.682	0.413	0.514	0.571	0.457	0.357	0.349	0.359	0.417
Yb (μg/g)	2.98	2.2	3.26	2.96	4.85	4.11	2.43	3	3.4	2.78	2.19	2.08	2.29	2.58
Lu (μg/g)	0.467	0.339	0.497	0.458	0.712	0.617	0.377	0.47	0.53	0.424	0.349	0.317	0.347	0.379
W (μg/g)	1.76	1.8	1.42	1.52	1.69	2.29	1.71	1.7	1.8	2.1	2.68	2.54	2.66	2.8
Re (μg/g)	0.006	0.004	0.005	0.008	0.011	0.009	0.007	0.01	0.01	0.013	0.017	0.017	0.018	0.018
Tl (μg/g)	1.33	1.18	1.28	1.3	1.58	1.3	1.44	1.72	1.65	2	2.31	2.28	2.3	2.38
Pb (μg/g)	15.2	20.5	22	18.8	27.3	19.4	30.6	31.2	25.2	20.6	19	16.6	20.8	20
Bi (μg/g)	0.269	0.27	0.286	0.264	0.371	0.376	0.369	0.445	0.58	0.519	0.508	0.477	0.466	0.57
Th (μg/g)	11.8	9.7	10.3	11	11.7	14.3	8.47	9.39	12	9.8	7.82	7.91	8	9.48
U (μg/g)	10.3	9.55	10.7	9.41	19.3	13.1	13.2	17	17.1	17.4	17.3	14.8	14.1	19.3
Nb (μg/g)	8.08	7.99	7.63	8.5	9.51	10.9	7.88	7.02	7.86	7.59	6.62	6.59	6.64	7.34
Ta (μg/g)	0.801	0.565	0.578	0.709	0.73	0.865	0.513	0.529	0.678	0.621	0.441	0.465	0.472	0.528
Zr (μg/g)	149	105	106	128	148	153	79.9	68.1	76.3	87.4	67.8	68.8	69.7	76.6
Hf (μg/g)	4.49	3.1	3.09	3.9	4.43	4.75	2.38	2.14	2.3	2.53	1.89	1.88	2.06	2.16
B (μg/g)	28.7	22.3	24.8	24.3	30.7	26.5	27.6	27.8	27.8	27.7	30.7	30.9	28.6	35.6

Supplementary Table S5: Carbon isotope data.

Sample No.	***1***	***2***	***3***	***4***	***5***	***6***	***7***	***8***	***9***	***10***	***11***	***12***	***13***	***14***
$\delta^{13}C_{V-PDB}$ (‰)	-5.4	-4.7	-5.2	-5.2	-5.9	-5.9	-5	-4.3	-4.1	-2.2	-0.7	-0.5	-0.9	-0.6
